# Semi-rational engineering of a thermostable aldo–keto reductase from *Thermotoga maritima* for synthesis of enantiopure ethyl-2-hydroxy-4-phenylbutyrate (EHPB)

**DOI:** 10.1038/s41598-017-03947-8

**Published:** 2017-06-21

**Authors:** Zhiguo Wang, Shuo Zhou, Shuangling Zhang, Sa Zhang, Fangmeng Zhu, Xiaolu Jin, Zhenming Chen, Xiaoling Xu

**Affiliations:** 1 Hangzhou Normal University, Hangzhou, Zhejiang 311121 China; 2 Apeloa Pharmaceutical Co., Ltd., Dongyang, Zhejiang 322118 China; 3 Yosemade Pharmaceutical Co., Ltd., Jinhua, Zhejiang 321025 China

## Abstract

A novel aldo-keto reductase Tm1743 characterized from *Thermotoga maritima* was explored as an effective biocatalyst in chiral alcohol production. Natural Tm1743 catalyzes asymmetric reduction of ethyl 2-oxo-4-phenylbutyrate (EOPB) at high efficiency, but the production of, ethyl (S)-2-hydroxy-4-phenylbutyrate ((S)-EHPB), which is less desirable, is preferred with an enantiomeric excess (*ee*) value of 76.5%. Thus, altering the enantioselectivity of Tm1743 to obtain the more valuable product (R)-EHPB for angiotensin drug synthesis is highly desired. In this work, we determined the crystal structure of Tm1743 in complex with its cofactor NADP^+^ at 2.0 Å resolution, and investigated the enantioselectivity of Tm1743 through semi-rational enzyme design. Molecular simulations based on the crystal structure obtained two binding models representing the *pro*-*S* and *pro*-*R* conformations of EOPB. Saturation mutagenesis studies revealed that Trp21 and Trp86 play important roles in determining the enantioselectivity of Tm1743. The best (R)- and (S)-EHPB preferring Tm1743 mutants, denoted as W21S/W86E and W21L/W118H, were identified; their *ee* values are 99.4% and 99.6% and the catalytic efficiencies are 0.81 and 0.12 mM^−1^s^−1^, respectively. Our work presents an efficient strategy to improve the enantioselectivity of a natural biocatalyst, which will serve as a guide for further exploration of new green catalysts for asymmetric reactions.

## Introduction

Chiral alcohols are one of the most important building blocks for many pharmaceutical intermediates and fine chemicals^1, 2^. Bio-catalytic production of chiral alcohols has received considerable attention during the last few decades, and several enzymes, including oxidoreductases (EC1), hydrolases (EC3) and lyases (EC4) have been found to produce chiral alcohols with remarkable chemo-, regio-, and stereoselectivity^3–6^. Aldo-keto reductases (AKRs) catalyze the NAD(P)H-dependent reduction of carbonyl groups to yield primary and secondary alcohols from a wide range of substrates, including aliphatic and aromatic aldehydes and ketones, ketoprostaglandins, ketosteroids and xenobiotics^3, 7–9^. There are more than 190 members of AKRs grouped in 16 families that are widely distributed in nature^10^. They are important enzymes in xylose metabolism in yeast^11^, vitamin C biosynthesis^12^, polyketide biosynthesis^13^ and steroid metabolism^14^. Human AKR enzymes play central roles in bio-activation or detoxication of drugs, carcinogens, and reactive aldehydes^15, 16^.

Several thermostable microbial AKRs have been extensively exploited in chiral alcohol production^2, 7, 8^ due to their stability at high temperatures and pressures as well as under high concentrations of chemical denaturants^17^. Recently, we identified a novel AKR enzyme, Tm1743, from the thermophile *Thermotoga maritima* ^18, 19^. This enzyme exhibits essential properties of an ideal biocatalyst, such as high thermostability, strong chemical tolerance and a broad substrate range with activity towards a series of ketones and aldehydes, the highest activity of which was observed at 90 °C and pH 9.0^18^. Coupled with NADPH-regeneration, Tm1743 has an *ee* value of 99.8% and conversion rate of 98% towards S-1-phenyl-2,2,2-trifluoroethanol, indicating its great potential in asymmetric synthesis of chiral alcohols^18^. However, the poor enantioselectivity of Tm1743 towards other substrates has greatly limited its application in the pharmaceutical industry. Therefore, improving and/or altering the enantioselectivity of Tm1743 for the synthesis of optically pure products has drawn our interest.

Here we report the successful enantioselectivity-oriented semi-rational engineering of Tm1743 through a combination of multiple approaches. In semi-rational engineering of enzymes, “smart” libraries of promising target residues that can significantly increase the efficiency of biocatalyst tailoring, are created based on prior knowledge of protein sequence, structure and function, as well as computational predictive algorithms^20^. In this work, ethyl 2-oxo-4-phenylbutyrate (EOPB), which is converted to ethyl (R)-2-hydroxy-4-phenylbutyrate ((R)-EHPB)^21, 22^, a key chiral intermediate for synthesis of the angiotensin converting enzyme inhibitors (ACEIs)^23^, was selected as a potent substrate. We demonstrated that Tm1743 indeed reduces EOPB to EHPB with 128% higher activity relative to ethyl-2-methylacetoacetate, but the preferred product is (S)-EOPB with an ee^s^ value of 76.5%. Using the solved X-ray crystal structure of Tm1743 in complex with NADP^+^, both binding modes of EOPB that leads to (R)- and (S)-EHPB products were obtained through molecular docking simulations. Based on the structural models and proposed catalytic mechanism, semi-rational engineering of Tm1743 was carried out, through which we effectively manipulated the enantioselectivity of Tm1743 to produce optically pure EHPB. Both (R)- and (S)-EHPB were obtained with *ee* values over 99%. In this work we illustrate the application of Tm1743 in producing chiral alcohols and demonstrate an efficient strategy to improve the enantioselectivity of a natural biocatalyst that can be applied in fine chemical and medicine synthesis.

## Results

### Overall architecture of Tm1743 in complex with NADP^+^

**Figure 1:**
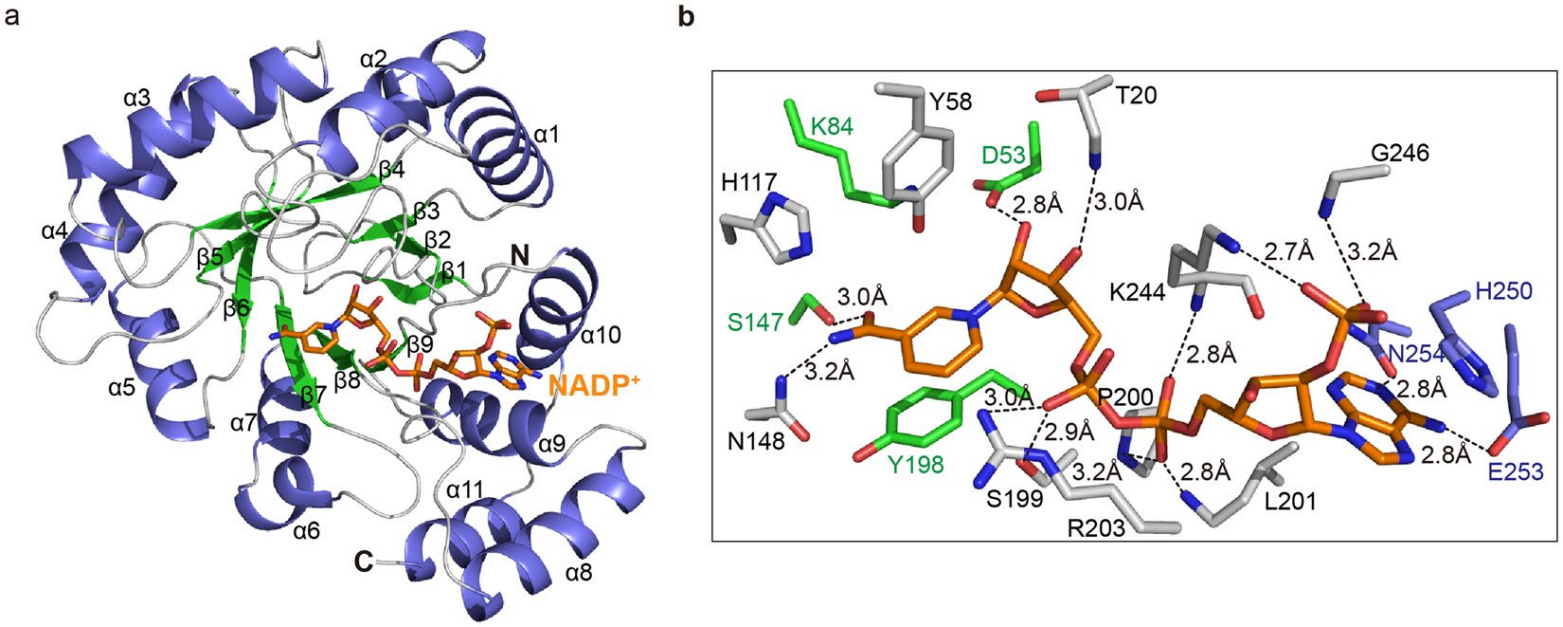
Overall crystal structure of Tm1743 in complex with NADP^+^. (**a**) Overall ribbon structure of Tm1743 in complex with NADP^+^. Secondary structures are colored in blue (α helices), green (β strands) and gray (coils), and cofactor NADP^+^ is shown as orange sticks. (**b**) Coordination of NADP^+^ in the cofactor binding pocket of Tm1743. Amino acids involved in NADP^+^ binding are shown as sticks and colored in accordance with their locations in the secondary structures. Hydrogen bonds mediating NADP^+^-Tm1743 interactions are indicated as dashed lines.

No structural characterization has been reported for Tm1743. In order to assist understanding of its catalytic mechanism, we crystallized Tm1743 in complex with the cofactor NADP^+^. The complex structure was determined by the molecular replacement method and refined to an *R*_*work*_ of 21.4% and *R*_*free*_ of 24.8% at 2.0 Å resolution (Table 1). The *P3*_*1*_ *21* trigonal crystal contains only one subunit of Tm1743 in the asymmetric unit. The refined structure covers the full-length enzyme (Met1-Gly274) and is composed of eleven α helices and nine β strands (Fig. 1a). Unlike typical AKR enzymes, Tm1743 lacks one β strand (Gly17-Gly19) in the core (β/α)_8_ barrel motif. Seven parallel β strands are surrounded by eight α helices in the outer layer, and a β hairpin formed by β1 and β2 caps the N-terminal end of the enzyme. Three additional α helices, α6 (Arg177-Asp182), α8 (Glu208-Gln220) and α11 (Glu264-Ser272), line the outside of the barrel (Fig. 1a). NADP^+^ was observed in the cofactor binding site of the structure. The nicotinamide and ribose ring of NADP^+^ is oriented towards the center of the β barrel, and the adenine ring is located in the cleft between α9 and α10 with its adjacent ribose ring and diphosphate groups sandwiched by the loops connecting α8-β8 and α10-β9 (Fig. 1a). The active site pocket occupies the space above the nicotinamide ring and is located in the center of the β barrel and surrounded by β3, β4, β5, β6 and connecting loops (Fig. 1a).

**Table 1:** X-ray diffraction data and structure refinement statistics.

Data Collection	
Cell parameters (Å)	*a* = *b* = 84.821, *c* = 93. 727
	α = β = 90°, γ = 120°
Space group	*P3*_*1*_ *21*
Resolution (Å)	50 (2.03)^*a*^–2.00
No. of all reflections	255930 (12490)
No. of unique reflections	25576 (1301)
Completeness (%)	95.8 (98.4)
Redundancy	10.0 (9.6)
I/σI	9.8 (3.3)
R_merge_^*b*^ (%)	10.9 (0.0)
Refinement	
Resolution (Å)	50.0–2.00
Total No. of reflections	25526
No. of reflections used	24240
*R*_*work*_/*R*_*free*_ (%)	21.4/24.8
No. of atoms	2438
Protein	2206
NADP^+^	48
Water	184
Wilson B-factors (Å^2^)	30.6
Average B-factors (Å^2^)	
Protein	34.5
NADP^+^	29.1
Water	44.0
R.m.s. deviations	
Bond lengths (Å)	0.009
Bond angle (°)	1.40
Ramachandran statistics	
Favored (%)	92.5
Allowed (%)	7.1
Generally allowed (%)	0.4
Outliers (%)	0.0

^a^Values in parentheses are for the highest resolution shell.

^b^ *R*_merge_ = ∑_*hkl*_ ∑_*i*_ │*I*_*i*_(*hkl*) −〈*I*(*hkl*)〉│/∑_*hkl*_ ∑_*i*_ *I*_*i*_(*hkl*), where *I*_*i*_(*hkl*) is the intensity of the *i*th measurement of reflection *hkl* and 〈*I*(*hkl*)〉 is the mean intensity of all symmetry-related reflections.

### Interaction of the cofactor NADP^+^ with Tm1743

In the structure (Fig. 1b), NADP^+^ is coordinated in the cofactor binding pocket formed by β6, β7 and β8 in an extended conformation. The nicotinamide ring stacks against the side chain of Tyr198, leaving the other side facing the catalytic tetrad D-Y-K-H (Asp53, Tyr58, Lys84, and His117) from the top (Fig. 1a and b). Hydrogen bond interactions are analyzed base on a commonly accepted criteria, i.e. the distance between hydrogen bond donor and acceptor atoms is less than 3.5 Å and the donor–hydrogen–acceptor angle is larger than 120°. The nicotinamide ring is stabilized by hydrogen bonds formed between the hydrogen atom of its amine group and the nitrogen atom of the Asn148 amide (3.2 Å), and its carbonyl oxygen with the hydroxyl hydrogen of Ser147 (3.0 Å). The nicotinamide ribose forms hydrogen bonds with the main chain nitrogen atom of Thr20 (3.0 Å) and hydroxyl hydrogen of Asp53 (2.8 Å). The free oxygen atoms of the pyrophosphate group are sandwiched by hydrogen bond interactions with the side chain hydrogens of Arg203 (3.0 Å) and Ser199 (2.9 Å), and the main chain nitrogens of Lys244 (2.8 Å), Leu201 (2.8 Å) and Pro200 (3.2 Å). The amine hydrogen of Lys244 forms a hydrogen bond with one oxygen of the 2’-phosphate group, and another oxygen of the same phosphate forms a hydrogen bond with Gly246 (3.2 Å). The adenine ring stacks in parallel with the imidazole ring of His250, and it is stabilized by hydrogen bond interactions with the carboxyl groups of Glu253 (2.8 Å) and Asn254 (2.8 Å) from α10. The ribose ring (adjacent to the adenine) is perpendicular to the adenine ring, with the solvent-exposed 2′-phosphate directed over the cofactor binding pocket (Fig. 1b).

### Molecular simulations of EOPB binding to Tm1743

Relative to the substrate ethyl-2-methylacetoacetate, Tm1743 demonstrated 128% higher activity in reducing EOPB to EHPB^18^. To investigate the mode of EOPB binding to Tm1743 and to identify the residues of Tm1743 that affect its enantioselectivity, molecular docking and molecular dynamics (MD) simulation were performed.

**Figure 2:**
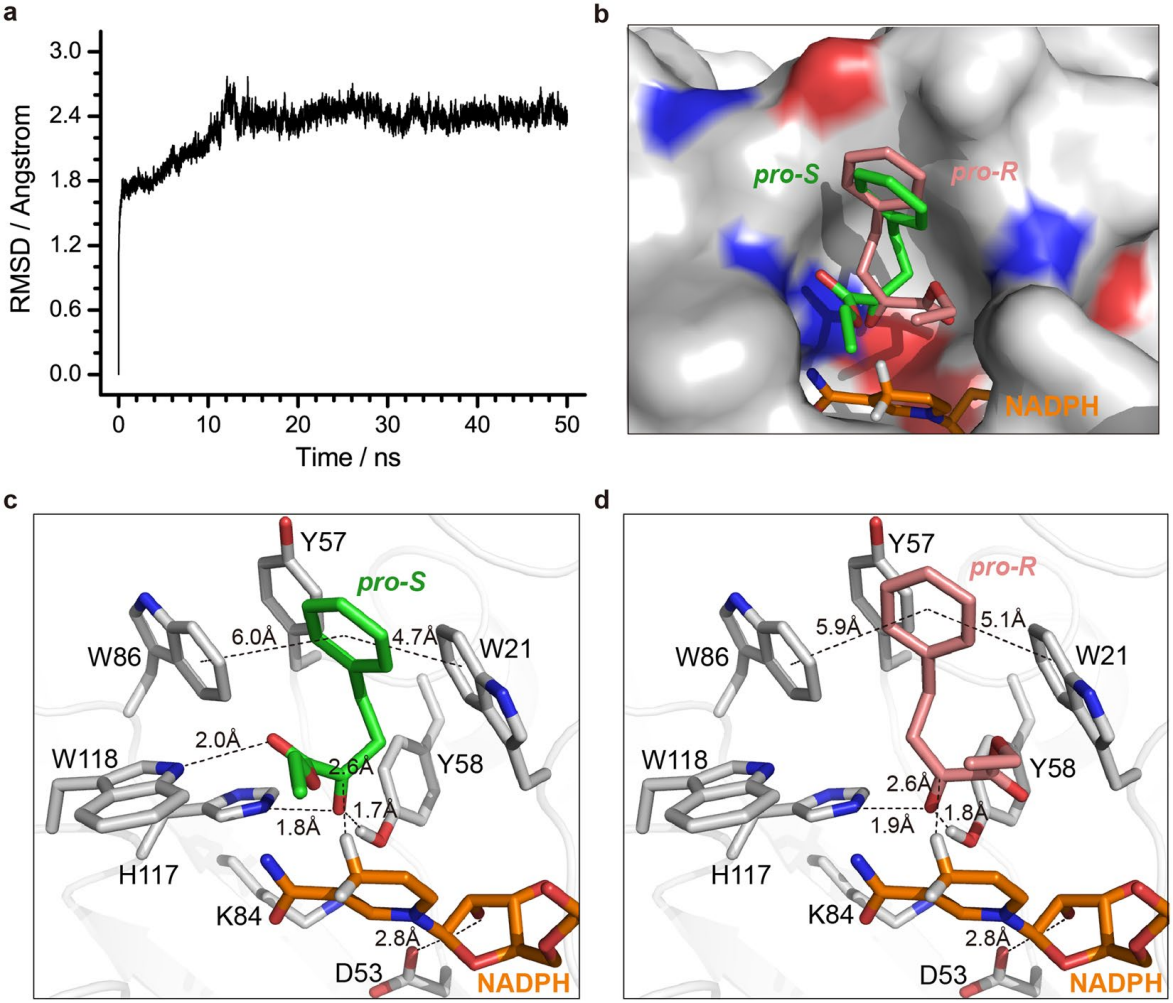
Molecular dynamic simulations of Tm1743 and docked conformations of Tm1743-NADPH-EOPB. (**a**) RMSD fluctuations of Tm1743 in the Tm1743-NADPH complex during the 50 ns MD simulation. (**b**) The *pro*-*S* (green) and *pro*-*R* (salmon) conformations of EOPB on the electrostatic surface of Tm1743. The positively charged, negatively charged and neutral amino acids are colored in blue, red, and gray, respectively. NADPH molecules are shown as orange sticks. (**c**) and (**d**) Stereo views of the *pro*-*S* (green) and *pro*-*R* (salmon) EOPB binding modes. Amino acids involved in EOPB coordination are shown as gray sticks.

No drastic conformational changes occurred during the MD process, as implied by the small fluctuations of root-mean-square deviation (RMSD) in Fig. 2a, indicating the complex structure of Tm1743-NADPH constructed based on the crystal structure of Tm1743-NADP^+^ was rather stable. However, two distinct conformations of Trp21 were observed through dihedral analysis, with its tryptophanyl group in the horizontal (φ > 80) and vertical orientation (φ < 70), respectively (φ defines the torsion angle formed by atoms CA-CB-CG-CD2 of Trp21) (Fig. S1). This is in accordance with the report that Trp20 of hALR2 functions as the catalytic sub-pocket switch through rotation of the indole moiety^24^.

The docking calculation yielded two ligand binding conformations named *pro*-*R*- and *pro*-*S*-EOPB, corresponding to the catalytic products (R)- and (S)-EHPB, respectively (Fig. 2b). In both conformations, the phenyl rings of EOPB have the same direction and are located in a hydrophobic pocket composed of the tyrosyl group of Tyr57 and the tryptophanyl groups of Trp21 and Trp86 (Fig. 2b‒d). However, the ester tails are in mirrored positions inside the substrate binding pocket. The ester group of *pro*-*S*-EOPB fits well in the cavity formed by Trp118, the nicotinamide ring of NADPH, and the active site residues His117, Tyr58 and Lys84 (Fig. 2b and c). In the *pro*-*R*-EOPB model, the hydrogen bond between Trp118 and the ester group of EOPB is disrupted, and the ester group fits in the pocket formed by Trp21, Tyr58 and the ribose ring of NADPH (Fig. 2b and d). Compared with *pro*-*S*-EOPB, the more hydrophobic environment around the ester tail of *pro*-*R*-EOPB may lead to relatively weaker binding with Tm1743.

### Proposed mechanism of EOPB reduction catalyzed by Tm1743

**Figure 3:**
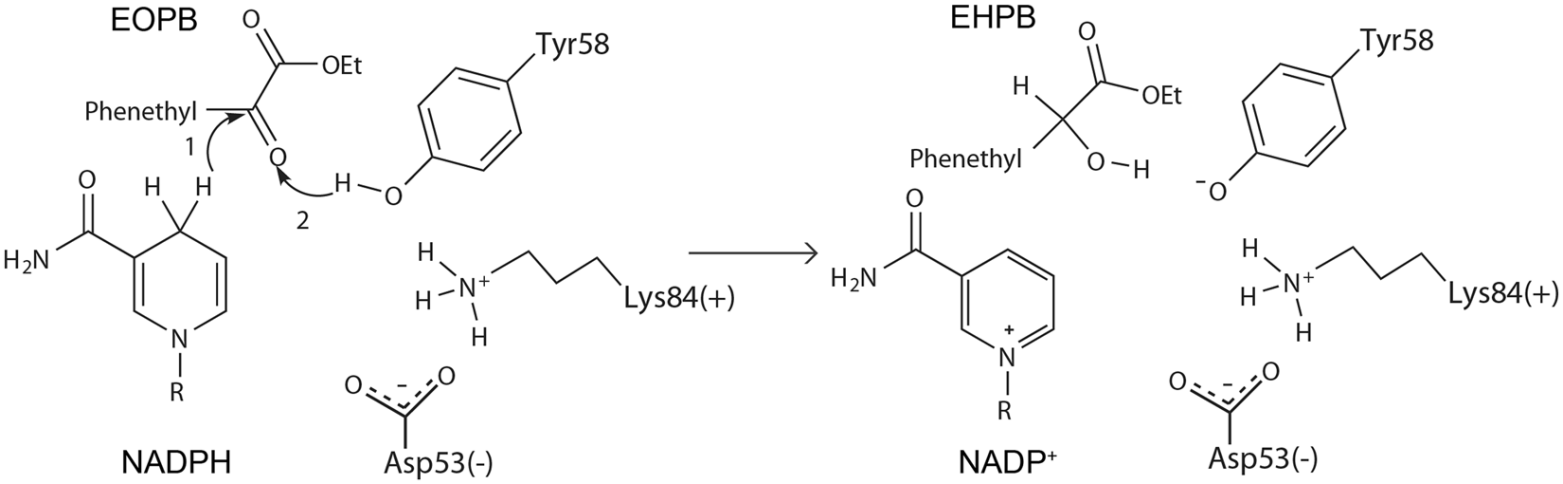
The proposed catalytic mechanism of Tm1743 reduction of EOPB to EHPB.

Previous studies of the kinetic characteristics of AKRs revealed that they follow an ordered bi−bi reaction mechanism, which is presumably a hallmark feature of all AKRs^24^. During the reductive transformation process, the 4-pro-R hydrogen of the NAD(P)H nicotinamide ring is transferred as a hydride directly to the *re* face of the substrate carbonyl carbon, and subsequently the carbonyl oxygen is protonated by a conserved tyrosine acting as a general acid^25–27^. Sequence alignment studies have indicated that the active-site region of AKRs is highly conserved, including the catalytic tetrad D-Y-K-H (Asp53, Tyr58, Lys84, and His117); Asp53 is present in 99%, Tyr58 in 97%, Lys84 in 97% and His117 in 88% of all annotated AKRs in the AKR database (www.med.upenn.edu/akr/). His117 is essential to maintaining the orientation of binding substrate; Tyr58, as the proton donor, acts as a general acid and is also essential to substrate binding; the Asp53^−^-Lys84^+^ pair can withdraw and delocalize the unbound electrons of the Tyr58 oxygen, making it easier to deprotonate, and thus lowering the pKa^24, 28^. Based on these roles, we propose that the reduction of EOPB is initiated by the attack of the hydride from NADPH nicotinamide ring towards the carbonyl carbon atom in EOPB. Next, the proton from the hydroxyl group of Tyr58 activated by the Asp53^−^-Lys84^+^ pair attacks the carbonyl oxygen of EOPB, thus promoting the conversion of EOPB to chiral EHPB (Fig. 3).

The enantioselectivity of Tm1743 is determined by the direction of the attack of hydride from the NADPH nicotinamide ring. In both the *pro*-*R*-EOPB and *pro*-*S*-EOPB conformations, the distances between the hydride of NADPH and the carbonyl carbon of EOPB are the same (2.6 Å). Compared with *pro*-*R*-EOPB, the carboxyl group of *pro*-*S*-EOPB forms one additional hydrogen bond with the amino of Trp118 (2.0 Å) (Fig. 2c and d), which further stabilizes the *pro*-*S*-EOPB conformation. This conformation is beneficial for attacking the *re* face of the carbonyl carbon, resulting in the production of (S)-EHPB. In contrast, the ester tail of *pro*-*R*-EOPB is flipped into the reverse direction, which facilitates the production of (R)-EHPB. Subsequently, the Asp53–Lys84^+^-activated hydroxyl hydrogen of Tyr58 attacks the carbonyl oxygen of EOPB (1.7 Å) and promotes the formation of (R)/(S)-EHPB (Figs 2c,d and 3).

### Saturation mutagenesis analyses of the enantioselectivity of Tm1743

Based on the catalytic mechanism and simulated models of Tm1743-NADPH-EOPB binding, the enantioselectivity of Tm1743 is affected by the coordination of EOPB in the substrate binding pocket. The higher binding stability of the *pro*-*S*-EOPB conformation and the (*S*)-preferred enantioselectivity of Tm1743 led us to propose that further increasing the binding stability of *pro*-*S*-EOPB may promote the preference of Tm1743 for the (*S*)-form. Alternatively, increasing the binding stability of *pro*-*R*-EOPB may increase Tm1743 preference for the (*R*)-form. Therefore, the residues involved in EOPB coordination were targeted for semi-rational design and an enantioselectivity study. Because Tyr57 mutants are tested to be inactive, Trp118, Trp86 and Trp21 were selected for saturation mutagenesis.

**Figure 4:**
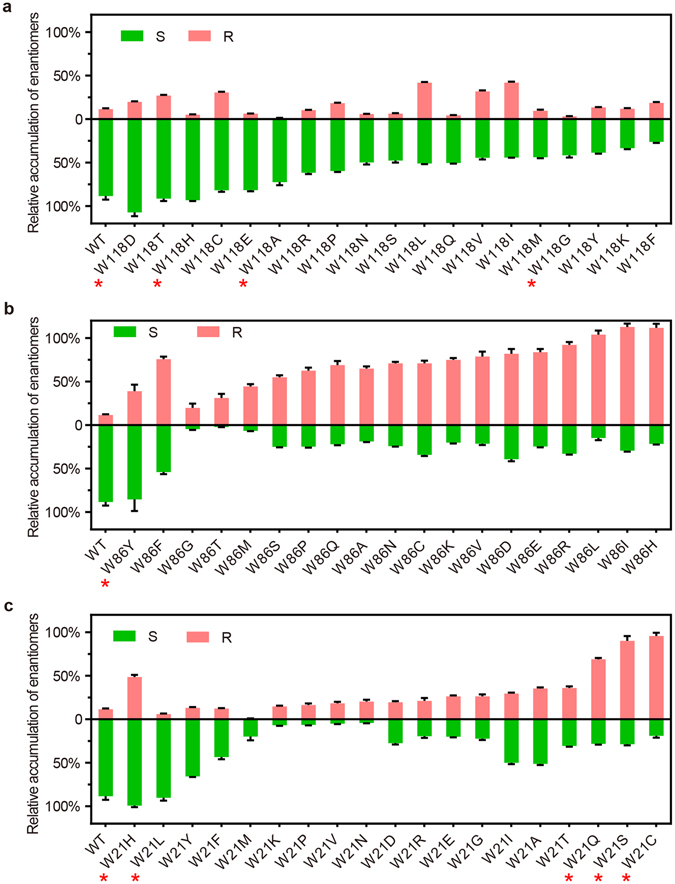
Investigation of the enantioselectivity of Tm1743 through saturation mutagenesis. The percentage of (S)-EHPB (green) and (R)-EHPB (salmon) produced by different saturation mutants of His118 (**a**), Trp86 (**b**) and Trp21 (**c**) are shown. The error bars represent the standard deviation from triplicate experiments. Important mutants with representative features are highlighted with red stars (*).

Primers used in site-directed mutagenesis are listed in Table S1. Saturation mutagenesis of Trp118 to 19 other amino acids did not change the overall enantioselectivity of Tm1743; a high percentage of (S)-EHPB was produced by most mutants. W118H, W118D, W118T, W118C and W118E mutants retained most of the enzyme activity and enantioselectivity of the wild-type enzyme, and W118H and W118D showed increased (S)-EHPB production. Substitution of Trp118 with other amino acids all decreased the enzyme activity but still maintained the (S)-EHPB preference. Notably, the catalytic product of W118A and W118G mutant was mostly (S)-EHPB, because the smaller side chains of these residues introduced a larger space for accommodating the ester group of *pro*-*S*-EOPB (Figs 4a and S2c). Interestingly, the W118L, W118V and W118I mutants showed increased (R)-EHPB production and slightly decreased enzyme activity (Fig. 4a), because introduction of neutral amino acids disrupted the hydrogen bond between Trp118 and *pro*-*S*-EOPB, which then destabilized the *pro*-*S*-EOPB conformation and promoted R-EHPB production. Taken together, these observations indicate that breaking the hydrogen bond between Trp118 and EOPB by mutagenesis does not affect the (S)-EHPB preference of Tm1743, but substitution of Trp118 with neutral amino acids enhances (R)-EHPB production.

The enantioselectivity of Tm1743 was dramatically altered when Trp86 was mutated, indicating Trp86 plays a critical role in determining the enantioselectivity of Tm1743 (Fig. 4b and Table S2). Except the W86Y mutant, the enantioslectivity of all other Trp86 mutants was completely altered, with a high percentage of (R)-EHPB being produced (Fig. 4b). In both the *pro*-*R*- *and pro*-*S*-EOPB models, the phenyl rings of EOPB stacked against the tryptophanyl side chain of Trp86 within 6 Å (Fig. 2c,d). Substitution of the indole ring of Trp86 with smaller side chains could eliminate the space constraints and provide enough space for multiple binding conformations of the phenyl ring, which in turn can direct the ester tail towards the *pro*-*R* orientation of EOPB. In contrast, introduction of a tyrosyl group at Trp86 might enhance its π-π interaction with Tyr57 and maintain the *pro*-*S* preference (Fig. S2c). Except for W86G, W86T and W86M, the Trp86 mutants had the same or higher enzyme activity than wild-type (Fig. 4b and Table S2). Because the interaction between the ester tail and catalytic tetrad is not affected in the *pro*-*R*-EOPB conformation (Fig. 2d), the mutants that produced more (R)-EHPB maintained similar enzyme activity as wild type. For the mutants W86G, W86T and W86M that showed low enzyme activity, the ester tail of EOPB may be directed into an orientation that is not beneficial to the reduction reaction.

Mutants where Trp21 was replaced with hydrophobic residues like Tyr, Phe and the neutral amino acids His, Leu and Met maintained (S)-EHPB preference (Fig. 4c and Table S2). Among these mutants, W21H showed increased enzyme activity, producing more (R)- and (S)-EHPB, because the imidazole ring of this mutant can provide similar steric hindrance as Trp21 to the phenyl ring of EOPB. However, replacement of Trp21 with charged amino acids like Lys, Arg, Glu, Asp, Asn and smaller amino acids like Pro, Val and Gly resulted in dramatically decreased enzyme activity, but more (R)-EHPB production (Fig. 4c). The polar and smaller amino acids at this position might decrease the π-π interactions between the phenyl rings of Trp21 and EOPB, destabilizing EOPB binding and result in low enzyme activity. However, the ester tail fits in the pocket formed by Trp21, Tyr58 and the ribose ring of NADPH (Fig. 2b and d). Thus, replacement of the indole ring with polar or smaller side chains produces a larger space for coordinating the ester group of *pro*-*R*-EOPB, giving rise to more possibilities to produce (R)-EHPB. Similarly, mutants with both a preference for (R)-EHPB and higher activity were obtained by mutating Trp21 to Gln, Ser and Cys (Fig. 4c).

### Promotion of enantioselectivity through combined mutagenesis

**Figure 5:**
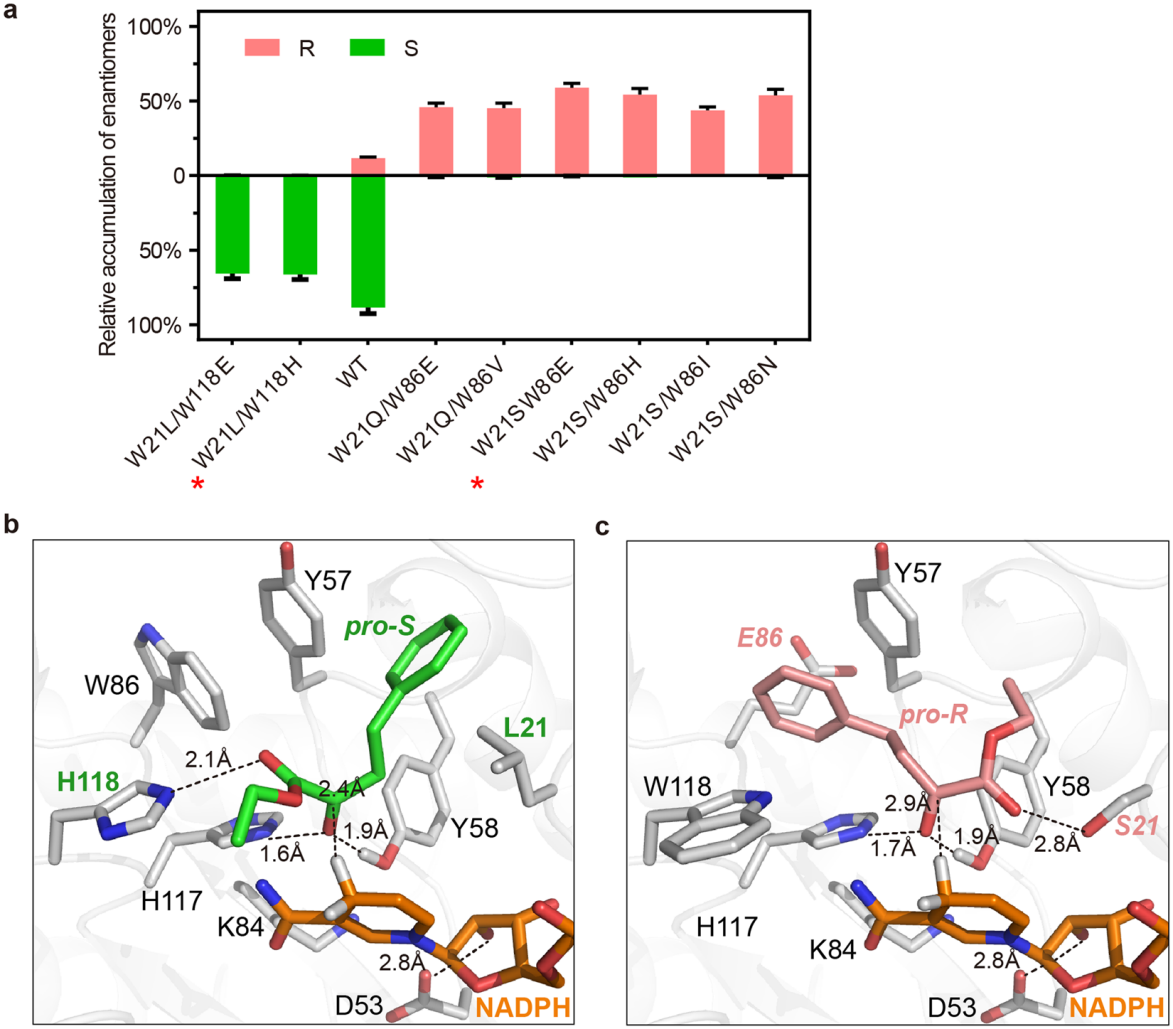
The optimal (S)-EHPB- and (R)-EHPB-preferring Tm1743 double mutants and models of their EOPB binding conformations. (**a**) The percentage of (S)-EHPB (green) and (R)-EHPB (salmon) produced by different Tm1743 double mutants. (**b**) and (**c**) Stereo views of the EOPB binding models of the best (S)-EHPB-preferring (W21L/W118H) and (R)-EHPB-preferring (W21S/W86E) mutants. Amino acids involved in EOPB coordination are shown as gray sticks, and the *pro*-*S* and *pro*-*R* conformations of EOPB are colored green and salmon, respectively. NADPH is shown as orange sticks.

To obtain an optimal biocatalyst that produces enantio pure chiral EHPB, the best single mutations were combined. The double mutants W21L/W118E and W21L/W118H displayed the best (S)-enantiomer selectivity, with *ee* values reaching 98.8% and 99.6% respectively (Fig. 5a and Table 2). Compared with wild-type Tm1743, the *K*_*m*_ of these two mutants for EOPB increased more than 10 fold, but the overall catalytic efficiency decreased (Table 3). Optimal (R)-enantiomer-preferring double mutants W21Q/W86E, W21Q/W86V, W21S/W86E, W21S/W86H, W21S/W86I and W21S/W86N were also obtained (Fig. 5a and Table 2). The W21S/W86E mutant showed the best (R)-enantiomer selectivity with an *ee* value of 99.4% (Table 2). The *K*_*m*_ of this mutant was comparable to wild-type Tm1743 (Table 3), but the relative activity decreased to 59.1% (Table 2). The binding conformations of EOPB with the best (S)- and (R)-EHPB-preferring mutants W21L/W118H and W21S/W86E were recomputed and are presented in Fig. 5b and c, respectively. By comparing with wild type (Fig. 2c and d), it is obvious that the phenyl group of *pro*-*S*-EOPB orients towards Leu21 in the hydrophobic pocket (Fig. 5b), whereas the phenyl group in *pro*-*R*-EOPB orients towards Glu86, and an additional hydrogen bond forms between the carboxyl oxygen of EOPB and the hydroxyl hydrogen of Ser21 (Fig. 5c). These results confirm that the double mutations are beneficial for enantioselectivity but are somewhat detrimental to the catalytic efficiency. Additionally, the thermal stabilities of the best double mutants were comparable to that of wild-type Tm1743 (Fig. S3), suggesting their bio-catalytic potential in the pharmaceutical industry.

**Table 2:** The enantioselectivity and relative activity of Tm1743 double mutants towards EOPB.

Mutants	*ee* ^*S*^ (%)	*ee* ^*R*^ (%)	Relative activity (%)
Wild-type	76.5 ± 0.1		100.0 ± 2.8
W21L/W118E	98.8 ± 0.1		66.0 ± 2.0
W21L/W118H	99.6 ± 0.1		66.3 ± 2.0
W21Q/W86E		96.8 ± 0.4	46.5 ± 1.5
W21Q/W86V		95. 3 ± 0.0	46.3 ± 2.0
W21S/W86E		99.4 ± 0.1	59.1 ± 1.7
W21S/W86H		95.7 ± 0.2	55.6 ± 2.3
W21S/W86I		96.4 ± 0.1	44.6 ± 1.2
W21S/W86N		97.6 ± 0.1	54.5 ± 2.3

**Table 3:** Kinetic parameters of the Tm1743 double mutants.

Enzymes	*K*_*m*_ (mM)	*k*_*cat*_ (s^−1^)	*k*_*cat*_/*K*_*m*_ (**s** ^−1^mM^−1^)
Wild-type	1.10 ± 0.04	1.96 ± 0.02	1.78 ± 0.03
W21L/W118E	10.53 ± 0.10	1.98 ± 0.02	0.19 ± 0.01
W21L/W118H	15.98 ± 0.20	1.98 ± 0.01	0.12 ± 0.01
W21S/W86E	1.25 ± 0.02	1.01 ± 0.01	0.81 ± 0.02
W21S/W86N	1.62 ± 0.02	0.84 ± 0.01	0.51 ± 0.02

## Discussion

The structure of Tm1743 in complex with NADP^+^ determined in this work reveals some exceptional features. Unlike typical AKR enzymes^25, 29^, the C-terminus of Tm1743 terminates with an α-helix (α11) instead of a long random coil. Additionally, the α4–β5 (His117–Pro124) and α8–β8 (Ser199–Leu206) connecting loops are remarkably short compared with other AKRs, resulting in a more compact secondary structure organization. These features contribute to the outstanding structural stability of Tm1743 at high temperatures.

Several studies have been performed to increase the catalytic activity or specificity of aldo-keto enzymes. Wang *et al*. demonstrated that the W28A mutation of aldo–keto reductase from *Lodderomyces elongisporus* results in higher molar conversion yields and lower *K*_*m*_ values^30^, W28 is the equivalent residue of Trp21 in Tm1743. Luo *et al*. and Penning *et al*. increased the catalytic activities of *Kluyveromyces lactis* aldo-keto reductase and 5 beta-reductase, respectively^31, 32^. Liu *et al*. found that conformational changes of Trp30 (the equivalent residue of Trp21) can increase the catalytic efficiency of AKR5C3^33^. A study by Zhu *et al*. showed that the W77M mutant has the largest effect on catalytic specificity of AKR7A5 towards succinic semialdehyde^34^, the side chain of W77 locates at the equivalent position of Trp 86. These studies provided important information that guided our mutagenesis study of Tm1743. However, to the best of our knowledge, the manipulation of aldo-keto reductase to increase enantioselectivity has not been reported previously.

In the current study, molecular docking simulations revealed that the EOPB phenyl group in the hydrophobic pocket stacks against the tyrosyl group of Tyr57 and between the tryptophanyl groups of Trp21 and Trp86 through π-π interactions^35, 36^ (Fig. 2c and d). Structural modeling indicates that Trp86, Tyr57 and Trp21 form a hydrophobic pocket that accommodates the *pro*-*S*- and *pro*-*R*-EOPBs in an energetically favorable manner. Saturation mutagenesis revealed that Trp86 and Trp21 have important roles in determining the enantioselectivity of Tm1743 during EOPB reduction. These two residues maintain a tight substrate binding pocket for EOPB through π-π interactions via their aromatic side chains^24^. Mutations of Trp86, and Trp21 to amino acids that increase the size of the hydrophobic pocket eliminates space constraints and facilitates the *pro*-*R* orientation of EOPB, and thus increases the production of (R)-EHPB. The W21L/W118H double mutation results in an open pocket that accommodates the *pro*-*S* orientation of the EOPB phenyl group (Fig. 5b). The W21S/W86E mutant *K*_*m*_ value for EOPB catalysis was comparable to wild-type Tm1743, probably due to the additional hydrogen bond provided by Ser21 (Fig. 5c and Table 3).

**Figure 6:**
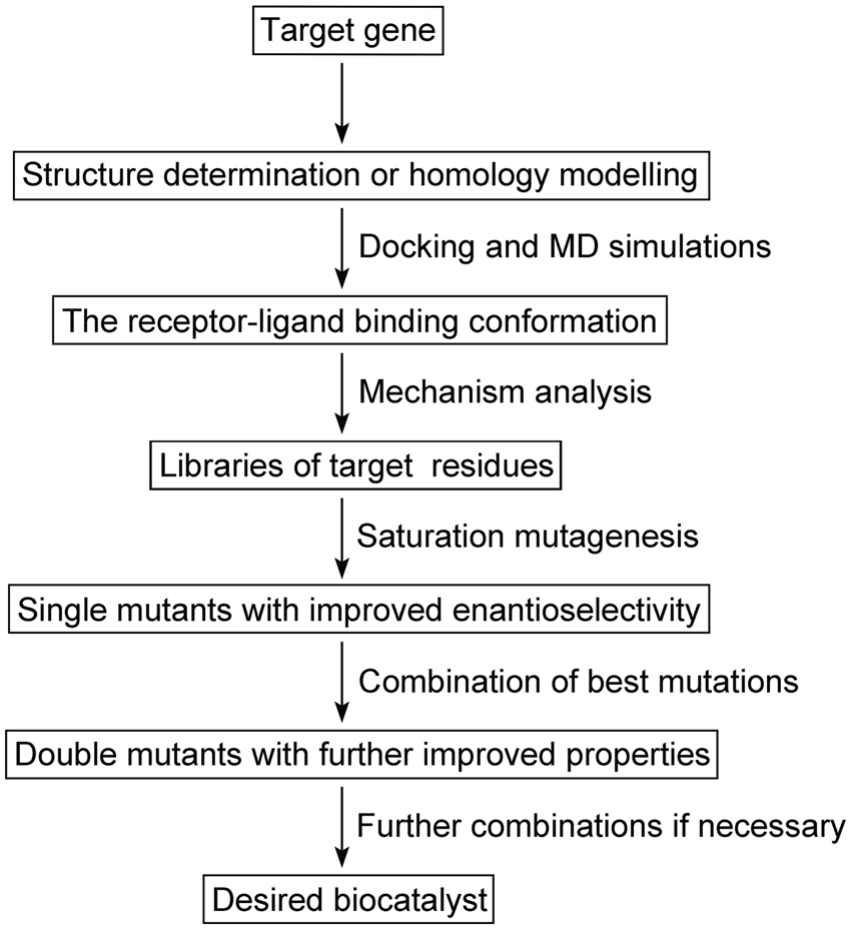
A flowchart of the semi-rational engineering of an enantioselective biocatalyst.

 Overall, we have established an efficient and environmentally-friendly method for the synthesis of enantiomerically pure EHPB by using the novel thermostable AKR enzyme Tm1743 (Fig. 6). Based on the Tm1743 crystal structure and molecular simulations, semi-rational enzyme engineering was performed with high efficiency. Mutants with high enantioselectivity and acceptable catalytic activity towards EOPB have been constructed; the optimal *ee* values for (R)- and (S)-EHPB are 99.60% and 99.40%, respectively. Our work is meaningful to the design and exploration of green catalysts for classic and novel asymmetric reactions.

## Methods

### Cloning, expression and purification

Plasmid pET-28a^18^ was used to express *Thermotoga maritima* Tm1743. The transformed *E*.*coli* BL21(DE3) cells were grown in 1 L LB medium containing 100 mg∙mL^−1^ Kanamycin at 37 °C until the OD_600_ reached 0.6–0.8. The culture was cooled, and protein expression was induced by addition of 0.1 mM isopropyl-*β*-D-thiogalactopyranoside (IPTG). The culture was then incubated overnight at 25 °C.

Cells were harvested by centrifugation at 4,596× g for 15 min at 4 °C. The harvested cells were resuspended in 40 mL distilled water and homogenized using a high-pressure homogenizer (Union, People’s Republic of China). Then the insoluble cell debris was removed by centrifugation at 34,541× g for 40 min at 4 °C. The supernatant containing crude soluble proteins was boiled at 100 °C for 10 min. After heating, the Tm1743 protein was still stable and remained soluble, but most of the *E*. *coli* proteins were precipitated. After centrifugation at 34,541× g at 4 °C for 20 min, the supernatant mainly containing Tm1743 protein was collected and diluted with an equal volume of binding buffer (25 mM Tris-HCl pH 8.5, 20 mM imidazole). The diluted supernatant was then loaded onto a Ni^2+^-chelating affinity chromatography column (2 mL, GE Healthcare, USA) and rinsed with 100 mL binding buffer to remove non-specifically bound proteins. The bound Tm1743 protein was eluted with elution buffer (25 mM Tris–HCl pH 8.5, 200 mM NaCl, 50–100 mM imidazole). The eluates were dialyzed against buffer containing 25 mM Tris–HCl pH 8.5, 150 mM NaCl and further purified using a HiLoad 16/600 Superdex 200 PG size exclusion column (GE Healthcare). A single absorption peak corresponding to the monomer of Tm1743 was observed. The homogeneous fractions were collected, and purity was verified by 15% SDS–PAGE analysis.

### Crystallization

The purified Tm1743 protein was concentrated to 30 mg∙mL^−1^ at 4 °C using an Amicon Ultra centrifugal filter device (10 kDa molecular-weight cutoff; Millipore). The protein concentration was determined using a NanoDrop device (Thermo Scientific, USA) by recording the absorption at 280 nm. The protein sample was diluted to 20 mg∙mL^−1^ in buffer (25 mM Tris–HCl pH 8.5 and 50 mM NaCl) and incubated with NADP^+^ at a 1:1.5 molar ratio before crystallization. Crystallization was performed in 24-well plates at 4 °C using the hanging-drop vapor-diffusion method. One μL protein sample was mixed with an equal volume of the reservoir solution (0.2 M calcium chloride dehydrate, 0.1 M sodium acetate trihydrate pH 4.6, 20% v/v 2-propanol), and the mixture was equilibrated against 200 μL reservoir solution.

### Crystal data collection, structure determination and refinement

The optimized Tm1743 crystals were cryo-protected by adding 25% glycerol to the reservoir solution and flash-freezing with liquid nitrogen. A 2.0-Å resolution data set was collected at −173 °C using an in-house X-ray source (Rigaku MicroMax-007 desktop rotating-anode X-ray generator with a Cu target operated at 40 kV and 30 mA) and an R-AXIS VI^++^ imaging-plate detector with a 130 mm crystal-to-detector distance at a wavelength of 1.54 Å. 180 diffraction frames were collected with 1° oscillation per image. The crystal belongs to space group *P3*_*1*_ *21* with unit cell dimensions *a* = 84.821 Å, *b* = 84.821 Å and *c* = 93.727 Å, α = β = 90°, γ = 120°.

Diffraction data were processed, integrated and scaled with HKL3000R software^37^. The data quality was assessed using SFCHECK^38^, and the solvent content was calculated using MATTHEWS_COEF from CCP4^39, 40^. Because Akr11a (PDB ID 1PYF) has 32% amino acid identity with Tm1743 and also uses NADPH as a cofactor, the Akr11a coordinates were used as a search model for molecular replacement. The PHASER program^41^ from the CCP4 package was employed to determine the initial phases. By deleting the non-aligned regions (Gly91-Pro100, Thr219-Lys247), we finally obtained the initial model of Tm1743. Iterative model building and refinement were performed using Coot^42^ and Refmac5^43^ to obtain the final model with an *R*_*work*_ of 21.4% and *R*_*free*_ of 24.8% at 2.0-Å resolution (Table 1).

### Molecular simulations

NADP^+^ was firstly converted to NADPH by adding hydrogen in Chimera software^44^. The Tm1743-NADPH complex was then immersed into the center of a truncated octahedron box of TIP3P water molecules with a margin distance of 12.0 Å, in which the crystal water molecules were retained. Na^+^ counterions were added to keep the system in neutrality. The complex was energy minimized by the steepest descent method for 2000 steps with the AMBER sander module^45^, during which Na^+^ ions and water molecules were set free while all residues of Tm1743 were restricted by a harmonic constraint of 100 kcal·mol^−1^Å^−2^. The system energy was further minimized by the conjugate gradient method for 5000 steps with no constraint on any component. Then system was gradually heated from 0 K to 300 K over a period of 200 ps under the NVT ensemble using a Langevin thermostat with a coupling coefficient of 1.0 ps. In this process a weak constraint of 10 kcal·mol^−1^Å^−2^ on the protein was imposed. The system was subsequently subjected to an equilibrium simulation for 200 ps by removing all constraints. Finally, a 50 ns production simulation was conducted using the NPT ensemble. During the whole process, the AMBER12 together with FF99SB force field that has been validated by many nanoseconds state-of-the-art MD simulations were used, and methods for treating the temperature and pressure and the parameters for calculating the electrostatic and van del Waals interactions were based on a previously reported procedure^46^. The time step was set to 2 fs, and the coordinates were saved every 1 ps to record the MD trajectory.

Molecular docking simulations were performed by using AutoDock (version 4.2.6)^47^. Non-polar hydrogen atoms of Tm1743 and EOPB were merged, and Gasteiger charges^48^ were computed. The binding region was defined by a 60 × 60 × 60 point grid box with 0.375 angstrom spacing between neighboring grid points, and the box center shared the same coordinates with the transferred hydride of NADPH. EOPB was obtained using GaussView software, and the structure was optimized by performing density functional theory calculations with Gaussian 03 software^49^ at the basis set level of 6–31 G(d). In docking computations, the Lamarckian genetic algorithm local search method with a medium energy evaluation of 2.5 × 10^6^ and the Solis and Wets algorithm with a maximum of 300 iterations were applied. A mutation rate of 0.02 and a crossover rate of 0.8 were used to generate new docking trials for subsequent generations. The elitism value was set to 1. The docking results from each of 150 independent calculations were clustered based on the root-mean square deviation (RMSD) between the Cartesian coordinates of ligand atoms and were ranked according to the binding free energy. The structures with relatively lower binding free energy and the most cluster members were chosen as potential binding conformations.

### Enzyme activity assays

Enzymatic activity was assayed spectrophotometrically at 35 °C by monitoring the decrease in the absorbance of NADPH at 340 nm (e = 6.22 mM^−1^cm^−1^). The standard assay mixture (1 mL) was composed of 50 mM potassium phosphate buffer (pH 6.5), 10 mM EOPB dissolved in 30 mM dimethyl sulfoxide (DMSO), 0.25 mM NADPH, and the appropriate amount of enzyme. One unit of enzymatic activity was defined as the amount of enzyme that catalyzes the oxidation of 1 μmol NADPH per min. Enzyme concentration was determined by the Bradford method. To obtain the kinetic parameters, *k*_*cat*_ and apparent Michaelis–Menten constant (*K*_*m*_), the enzymatic activity assay was performed under the above standard conditions with EOPB concentrations of 0.25 mM, 0.5 mM, 1 mM, 2 mM, 4 mM, 8 mM, and 16 mM. The *k*_*cat*_ and *K*_*m*_ values were determined by the non-linear least squares fitting method.

### Enantiomeric evaluation of Tm1743 mutants

Asymmetric reduction of EOPB was conducted in a 2 mL reaction mixture composed of 0.5 mM NADPH, 30 mM EOPB, an appropriate amount of enzyme, 1 U formate dehydrogenase, and 250 mM sodium formate in 200 mM potassium phosphate buffer (pH 7.2) at 35 °C for 15 h. The conversion and enantiomeric excess were determined by high performance liquid chromatography (HPLC, Waters). The products were extracted twice with n-hexane, and then the extracts were loaded on a Chiracel-OD-H column (25.9 × 0.46 cm, Daicel, Japan) and detected by HPLC at 230 nm. A hexane/isopropanol mixture (97:3, v/v) was used as the mobile phase with a flow rate of 1 mL**∙**min^−1^. The retention times of EOPB, (S)-EHPB, and (R)-EHPB were 17.5, 12.5, and 13.1 min respectively.

### Thermal stability of mutant enzymes

To investigate thermal stability, different enzyme mutants were kept at temperatures ranging from 50 °C to 85 °C in 50 mM potassium phosphate buffer (pH 6.5) for 1 h. Standard enzyme activity assays were carried out to measure residual activity, which was expressed as a percentage of the initial activity without any pre-incubation. Assays were performed with a reaction mixture (3 mL) containing 3 mM EOPB, 30 mM DMSO (to dissolve EOPB), 50 mM Tris-HCl buffer (pH 9.0), 12 mM NADPH, and an appropriate amount of enzyme, and were incubated at a specific temperature for 3 min. Specific activity (unit U∙mg^−1^) was plotted against the temperature to show the temperature dependence of the enzyme activity.

## Electronic supplementary material

Supplementary information
