# Supplementary material for: Semi-rational engineering of a thermostable aldo–keto reductase from Thermotoga maritima for synthesis of enantiopure ethyl-2-hydroxy-4-phenylbutyrate (EHPB)
Source: Sci Rep. 2017 Jun 21;7:4007. doi: 10.1038/s41598-017-03947-8 (PMC5479831; doi:10.1038/s41598-017-03947-8)
Supplement: Supplementary file 1 — Supplementary information [file 41598_2017_3947_MOESM1_ESM.doc]

**Semi-rational engineering of a thermostable aldo–keto reductase from *Thermotoga maritima* for synthesis of enantiopure ethyl-2-hydroxy-4-phenylbutyrate (EHPB)**

Zhiguo Wang,^a,†^ Shuo Zhou,^a,†^ Shuangling Zhang,^a^ Sa Zhang,^a^ Fangmeng Zhu,^b^ Xiaolu Jin,^c^ Zhenming Chen,^a,#^ Xiaoling Xu^a,#^

Hangzhou Normal University, Hangzhou, Zhejiang 311121, China^a^

Apeloa Pharmaceutical Co., Ltd., Dongyang, Zhejiang 322118, China^b^

Yosemade Pharmaceutical Co., Ltd., Jinhua, Zhejiang 321025, China^c^

^†^Z. W. and S. Z. contributed equally to this work

^#^Corresponding authors:

Xiaoling Xu, e-mail: xuxl@hznu.edu.cn, tel: 86-571-28861723

Zhenming Chen, e-mail: zchenhznu@126.com, tel: 86-571-28869373

**Mutant construction, expression and purification**

The Site-Directed Mutagenesis was achieved by using QuikChange Lightning Site-Directed Mutagenesis Kits (Agilent), pET-28a plasmid containing native *Thermotoga maritima* Tm1743 was used as the template. Then plasmid containing mutant Tm1743 was transformed into Escherichia coli BL21(DE3) cells (Novagen, USA) for mutant enzyme expression. The transformed cells were grown in 1 L LB medium containing 100 mg∙mL^-1^ Kanamycin at 310 K until the OD600 reached 0.6~0.8. The culture was cooled and induced with 0.1 mM isopropyl-β-D-thiogalactopyranoside (IPTG) and incubated overnight at 298 K.

Cells were harvested by centrifugation at 4,596×g and 277 K for 15 min. The harvested cells were re-suspended in 40 mL distilled water and homogenized using a high-pressure homogenizer (Union, People’s Republic of China). Then the insoluble cell debris was removed by centrifugation at 34,541×g for 40 min at 277 K. Because the thermal stability of mutant was unclear, heating treatment was eliminated. The supernatant containing mutated Tm1743 enzyme was collected and diluted by equal volume of binding buffer (25 mM Tris-HCl pH 8.5, 20 mM imidazole). The diluted supernatant was then loaded onto a Ni^2+^-chelating affinity chromatography column (GE Healthcare, USA) and rinsed with 100 mL binding buffer to remove non-specifically bound proteins. The bound enzyme was eluted with elution buffer (25 mM Tris–HCl pH 8.5, 200 mM NaCl, 50~100 mM imidazole). The eluates were dialyzed against the buffer containing 25 mM Tris–HCl pH 8.5, 150 mM NaCl and further purified by using a size exclusion column HiLoad 16/600 Superdex 200 PG (GE Healthcare).

The QuikChange Primer Design program was applied to design the mutagenic primers, which were listed as follows. Because the double mutations were achieved through two successive single mutations, therefore the primers for double mutants were not presented.

**Tables**

**Table S1.** Primers used in site-directed mutagenesis.

| Mutants | Primers |
| --- | --- |
| W118A | 5'-GGGATTTCCGGATTCGGCGCGTGAATCAGGTACAGATC-3'  5'-GATCTGTACCTGATTCACGCGCCGAATCCGGAAATCCC-3' |
| W118C | 5'-GGATTTCCGGATTCGGGCAGTGAATCAGGTACA-3'  5'-TGTACCTGATTCACTGCCCGAATCCGGAAATCC-3' |
| W118D | 5'-CAGCGGGATTTCCGGATTCGGATCGTGAATCAGGTACAGATCAAC-3'  5'-GTTGATCTGTACCTGATTCACGATCCGAATCCGGAAATCCCGCTG-3' |
| W118E | 5'-CAGATGGGTCGGCTCAACTTTGCTCACAATGAACAGATCT-3'  5'-GATCTGTACCTGATTCACGAGCCGAATCCGGAAATCCC-3' |
| W118F | 5'-GATTTCCGGATTCGGGAAGTGAATCAGGTACAGATCAACATAATCC-3'  5'-GGATTATGTTGATCTGTACCTGATTCACTTCCCGAATCCGGAAATC-3' |
| W118G | 5'-ATTTCCGGATTCGGCCCGTGAATCAGGTACAGAT-3'  5'-ATCTGTACCTGATTCACGGGCCGAATCCGGAAAT-3' |
| W118H | 5'-CAGCGGGATTTCCGGATTCGGATGGTGAATCAGGTACAGATCAAC-3'  5'-GTTGATCTGTACCTGATTCACCATCCGAATCCGGAAATCCCGCTG-3' |
| W118I | 5'-CAGCGGGATTTCCGGATTCGGTATGTGAATCAGGTACAGATCAAC-3'  5'-GTTGATCTGTACCTGATTCACATACCGAATCCGGAAATCCCGCTG-3' |
| W118K | 5'-GGGATTTCCGGATTCGGCTTGTGAATCAGGTACAGATC-3'  5'-GATCTGTACCTGATTCACAAGCCGAATCCGGAAATCCC-3' |
| W118L | 5'-TCCGGATTCGGCAAGTGAATCAGGTACAGATCAACA-3'  5'-TGTTGATCTGTACCTGATTCACTTGCCGAATCCGGA-3' |
| W118M | 5'-GGGATTTCCGGATTCGGCATGTGAATCAGGTACAGATC-3'  5'-GATCTGTACCTGATTCACATGCCGAATCCGGAAATCCC-3' |
| W118N | 5'-CAGCGGGATTTCCGGATTCGGATTGTGAATCAGGTACAGATCAAC-3'  5'-GTTGATCTGTACCTGATTCACAATCCGAATCCGGAAATCCCGCTG-3' |
| W118P | 5'-GATCTGTACCTGATTCACCCGCCGAATCCGGAAATCCC-3'  5'-GGGATTTCCGGATTCGGCGGGTGAATCAGGTACAGATC-3' |
| W118Q | 5'-GATCTGTACCTGATTCACCAGCCGAATCCGGAAATCCC-3'  5'-GGGATTTCCGGATTCGGCTGGTGAATCAGGTACAGATC-3' |
| W118R | 5'-ATCTGTACCTGATTCACAGGCCGAATCCGGAAAT-3'  5'-ATTTCCGGATTCGGCCTGTGAATCAGGTACAGAT-3' |
| W118S | 5'-CCGGATTCGGCGAGTGAATCAGGTACAGATCAA-3'  5'-TTGATCTGTACCTGATTCACTCGCCGAATCCGG-3' |
| W118T | 5'-GGGATTTCCGGATTCGGCGTGTGAATCAGGTACAGATC-3'  5'-GATCTGTACCTGATTCACACGCCGAATCCGGAAATCCC-3' |
| W118V | 5'-GGGATTTCCGGATTCGGCACGTGAATCAGGTACAGATC-3'  5'-GATCTGTACCTGATTCACGTGCCGAATCCGGAAATCCC-3' |
| W118Y | 5'-GATTTCCGGATTCGGATAGTGAATCAGGTACAGATCAACATAATCC-3'  5'-GGATTATGTTGATCTGTACCTGATTCACTATCCGAATCCGGAAATC-3' |
| W86A | 5'-CAGATGGGTCGGCGCAACTTTGCTCACAATGAACAGATCT-3'  5'-AGATCTGTTCATTGTGAGCAAAGTTGCGCCGACCCATCTG-3' |
| W86C | 5'-CGCAGATGGGTCGGGCAAACTTTGCTCACAAT-3'  5'-ATTGTGAGCAAAGTTTGCCCGACCCATCTGCG-3' |
| W86D | 5'-ACGCAGATGGGTCGGATCAACTTTGCTCACAATGAACAGATCTTCGC-3'  5'-GCGAAGATCTGTTCATTGTGAGCAAAGTTGATCCGACCCATCTGCGT-3' |
| W86E | 5'-CAGATGGGTCGGCTCAACTTTGCTCACAATGAACAGATCT-3'  5'-AGATCTGTTCATTGTGAGCAAAGTTGAGCCGACCCATCTG-3' |
| W86F | 5'-GCAGATGGGTCGGGAAAACTTTGCTCACAATGAACAGATC-3'  5'-GATCTGTTCATTGTGAGCAAAGTTTTCCCGACCCATCTGC-3' |
| W86G | 5'-GATGGGTCGGCCCAACTTTGCTCACAATGAACAGAT-3'  5'-ATCTGTTCATTGTGAGCAAAGTTGGGCCGACCCATC-3' |
| W86H | 5'-ACGCAGATGGGTCGGATGAACTTTGCTCACAATGAACAGATCTTCGC-3'  5'-GCGAAGATCTGTTCATTGTGAGCAAAGTTCATCCGACCCATCTGCGT-3' |
| W86I | 5'-ACGCAGATGGGTCGGTATAACTTTGCTCACAATGAACAGATCTTCGC-3'  5'-GCGAAGATCTGTTCATTGTGAGCAAAGTTATACCGACCCATCTGCGT-3' |
| W86K | 5'-CAGATGGGTCGGCTTAACTTTGCTCACAATGAACAGATCT-3'  5'-AGATCTGTTCATTGTGAGCAAAGTTAAGCCGACCCATCTG-3' |
| W86L | 5'-AGATGGGTCGGCAAAACTTTGCTCACAATGAACAGA-3'  5'-TCTGTTCATTGTGAGCAAAGTTTTGCCGACCCATCT-3' |
| W86M | 5'-CAGATGGGTCGGCATAACTTTGCTCACAATGAACAGATCT-3'  5'-AGATCTGTTCATTGTGAGCAAAGTTATGCCGACCCATCTG-3' |
| W86N | 5'-ACGCAGATGGGTCGGATTAACTTTGCTCACAATGAACAGATCTTCGC-3'  5'-GCGAAGATCTGTTCATTGTGAGCAAAGTTAATCCGACCCATCTGCGT-3' |
| W86P | 5'-CAGATGGGTCGGCGGAACTTTGCTCACAATGAACAGATCT-3'  5'-AGATCTGTTCATTGTGAGCAAAGTTCCGCCGACCCATCTG-3' |
| W86Q | 5'-GATGGGTCGGCCTAACTTTGCTCACAATGAACAGAT-3'  5'-ATCTGTTCATTGTGAGCAAAGTTAGGCCGACCCATC-3' |
| W86R | 5'-GATGGGTCGGCCTAACTTTGCTCACAATGAACAGAT-3'  5'-ATCTGTTCATTGTGAGCAAAGTTAGGCCGACCCATC-3' |
| W86S | 5'-AGATGGGTCGGCGAAACTTTGCTCACAATGAACAGA-3'  5'-TCTGTTCATTGTGAGCAAAGTTTCGCCGACCCATCT-3' |
| W86T | 5'-CAGATGGGTCGGCGTAACTTTGCTCACAATGAACAGATCT-3'  5'-AGATCTGTTCATTGTGAGCAAAGTTACGCCGACCCATCTG-3' |
| W86V | 5'-CAGATGGGTCGGCACAACTTTGCTCACAATGAACAGATCT-3'  5'-AGATCTGTTCATTGTGAGCAAAGTTGTGCCGACCCATCTG-3' |
| W86Y | 5'-GCAGATGGGTCGGATAAACTTTGCTCACAATGAACAGATC-3'  5'-GATCTGTTCATTGTGAGCAAAGTTTATCCGACCCATCTGC-3' |
| W21A | 5'-ACCGCCGATACCCGCGGTACCCAGACCC-3'  5'-GGGTCTGGGTACCGCGGGTATCGGCGGT-3' |
| W21C | 5'-AACCGCCGATACCGCAGGTACCCAGAC-3'  5'-GTCTGGGTACCTGCGGTATCGGCGGTT-3' |
| W21D | 5'-TTCAAAACCGCCGATACCATCGGTACCCAGACCCAGTGC-3'  5'-GCACTGGGTCTGGGTACCGATGGTATCGGCGGTTTTGAA-3' |
| W21E | 5'-AAACCGCCGATACCCTCGGTACCCAGACCCAG-3'  5'-CTGGGTCTGGGTACCGAGGGTATCGGCGGTTT-3' |
| W21F | 5'-ACCGCCGATACCGAAGGTACCCAGACCCAG-3'  5'-CTGGGTCTGGGTACCTTCGGTATCGGCGGT-3' |
| W21G | 5'-CGCCGATACCCCCGGTACCCAGACC-3'  5'-GGTCTGGGTACCGGGGGTATCGGCG-3' |
| W21H | 5'-TTCAAAACCGCCGATACCATGGGTACCCAGACCCAGTGC-3'  5'-GCACTGGGTCTGGGTACCCATGGTATCGGCGGTTTTGAA-3' |
| W21I | 5'-TTCAAAACCGCCGATACCTATGGTACCCAGACCCAGTGC-3'  5'-GCACTGGGTCTGGGTACCATAGGTATCGGCGGTTTTGAA-3' |
| W21K | 5'-AAACCGCCGATACCCTTGGTACCCAGACCCAG-3'  5'-CTGGGTCTGGGTACCAAGGGTATCGGCGGTTT-3' |
| W21L | 5'-GCCGATACCCAAGGTACCCAGACCCAGTG-3'  5'-CACTGGGTCTGGGTACCTTGGGTATCGGC-3' |
| W21M | 5'-AAACCGCCGATACCCATGGTACCCAGACCCAG-3'  5'-CTGGGTCTGGGTACCATGGGTATCGGCGGTTT-3' |
| W21N | 5'-TTCAAAACCGCCGATACCATTGGTACCCAGACCCAGTGC-3'  5'-GCACTGGGTCTGGGTACCAATGGTATCGGCGGTTTTGAA-3' |
| W21P | 5'-ACCGCCGATACCCGGGGTACCCAGACCC-3'  5'-GGGTCTGGGTACCCCGGGTATCGGCGGT-3' |
| W21Q | 5'-AAACCGCCGATACCCTGGGTACCCAGACCCAG-3'  5'-CTGGGTCTGGGTACCCAGGGTATCGGCGGTTT-3' |
| W21R | 5'-CGCCGATACCCCTGGTACCCAGACC-3'  5'-GGTCTGGGTACCAGGGGTATCGGCG-3' |
| W21S | 5'-CCGATACCCGAGGTACCCAGACCCAG-3'  5'-CTGGGTCTGGGTACCTCGGGTATCGG-3' |
| W21T | 5'-ACCGCCGATACCCGTGGTACCCAGACCC-3'  5'-GGGTCTGGGTACCACGGGTATCGGCGGT-3' |
| W21V | 5'-AAACCGCCGATACCCACGGTACCCAGACCCAG-3'  5'-CTGGGTCTGGGTACCGTGGGTATCGGCGGTTT-3' |
| W21Y | 5'-AAACCGCCGATACCATAGGTACCCAGACCCAGTG-3'  5'-CACTGGGTCTGGGTACCTATGGTATCGGCGGTTT-3' |

**Table S2.** The enantioselectivity and relative activity of single mutants of Tm1743 towards EOPB^a^

| Mutant | ee^S^ (%) | ee^R^ (%) | Relative activity (%) |
| --- | --- | --- | --- |
| WT | 76.5 ± 0.1 |  | 100.0 ± 2.8 |
| W118D | 68.7 ± 0.3 |  | 127.0 ± 5.0 |
| W118T | 54.1 ± 0.1 |  | 118.5 ± 3.6 |
| W118H | 89.4 ± 0.7 |  | 98.3 ± 1.2 |
| W118C | 45.4 ± 2.5 |  | 112.5 ± 2.5 |
| W118E | 85.6 ± 1.8 |  | 87.7 ± 1.5 |
| W118A | 96.5 ± 1.9 |  | 73.6 ± 3.6 |
| W118R | 70.9 ± 0.4 |  | 72.0 ± 1.7 |
| W118P | 52.5 ± 0.3 |  | 78.1 ± 1.5 |
| W118N | 78.6 ± 0.2 |  | 55.7 ± 2.6 |
| W118S | 76.1 ± 0.5 |  | 53.9 ± 2.9 |
| W118L | 9.8 ± 0.3 |  | 92.9 ± 1.5 |
| W118Q | 83.3 ± 0.4 |  | 54.8 ± 1.0 |
| W118V | 16.5 ± 0.3 |  | 76.2 ± 3.2 |
| W118I | 2.3 ± 0.8 |  | 86.3 ± 1.2 |
| W118M | 63.6 ± 3.9 |  | 53.5 ± 1.1 |
| W118G | 85.8 ± 0.6 |  | 44.7 ± 2.9 |
| W118Y | 47.7 ± 0.3 |  | 52.2 ± 1.5 |
| W118K | 46.5 ± 0.5 |  | 45.6 ± 1.5 |
| W118F | 16.4 ± 0.3 |  | 44.8 ± 1.6 |
| W86Y | 37.4 ± 1.5 |  | 124.3 ± 9.9 |
| W86F |  | 17.0 ± 0.5 | 129.8 ± 5.2 |
| W86G |  | 63.3 ± 0.5 | 24.4 ± 5.7 |
| W86T |  | 87.8 ± 0.7 | 33.3 ± 5.0 |
| W86M |  | 74.4 ± 0.4 | 51.0 ± 2.9 |
| W86S |  | 37.5 ± 1.7 | 80.0 ± 2.5 |
| W86P |  | 43.4 ± 0.4 | 87.3 ± 4.5 |
| W86Q |  | 51.7 ± 0.6 | 91.0 ± 5.7 |
| W86A |  | 55.2 ± 0.1 | 83.8 ± 3.2 |
| W86N |  | 49.2 ± 0.8 | 95.2 ± 1.9 |
| W86C |  | 35.3 ± 0.3 | 105.2 ± 4.4 |
| W86K |  | 57.8 ± 2.5 | 95.1 ± 1.7 |
| W86V |  | 57.6 ± 0.5 | 100.2 ± 7.1 |
| W86D |  | 35.5 ± 0.3 | 121.0 ± 8.0 |
| W86E |  | 54.9 ± 0.1 | 108.4 ± 4.6 |
| W86R |  | 47.4 ± 0.5 | 125.3 ± 4.0 |
| W86L |  | 75.2 ± 3.1 | 118.8 ± 6.8 |
| W86I |  | 58.8 ± 0.5 | 142.2 ± 5.0 |
| W86H |  | 67.7 ± 0.6 | 133.4 ± 5.1 |
| W21L | 87.2 ± 1.5 |  | 96.3 ± 3.0 |
| W21Y | 66.4 ± 1.6 |  | 78.7 ± 1.1 |
| W21F | 54.9 ± 2.7 |  | 55.9 ± 2.5 |
| W21M | 95.5 ± 4.9 |  | 20.2 ± 5 |
| W21K |  | 37.9 ± 3.2 | 21.7 ± 1.5 |
| W21P |  | 44.9 ± 1.2 | 22.8 ± 2.5 |
| W21V |  | 57.5 ± 1.9 | 23.5 ± 2.0 |
| W21N |  | 66.6 ± 1.2 | 24.5 ± 2.5 |
| W21D | 16.1 ± 2.5 |  | 47.1 ± 2.5 |
| W21R |  | 4.8 ± 2.5 | 40.7 ± 5.0 |
| W21E |  | 14.1 ± 1.2 | 46.5 ± 1.5 |
| W21G |  | 9.0 ± 0.7 | 48.5 ± 4.0 |
| W21I | 25.3 ± 0.2 |  | 79.7 ± 2.5 |
| W21A | 18.1 ± 0.5 |  | 86.5 ± 2.6 |
| W21H | 34.1 ± 2.4 |  | 147.9 ± 2.3 |
| W21T |  | 8.4 ± 1.1 | 66.8 ± 2.5 |
| W21Q |  | 42.1 ± 1.3 | 97.2 ± 2.0 |
| W21S |  | 52.1 ± 1.0 | 118.7 ± 6.8 |
| W21C |  | 67.2 ± 3.8 | 114.8 ± 3.0 |

*^a^* The standard errors were calculated from triplicate experiments.

**Figures**

**
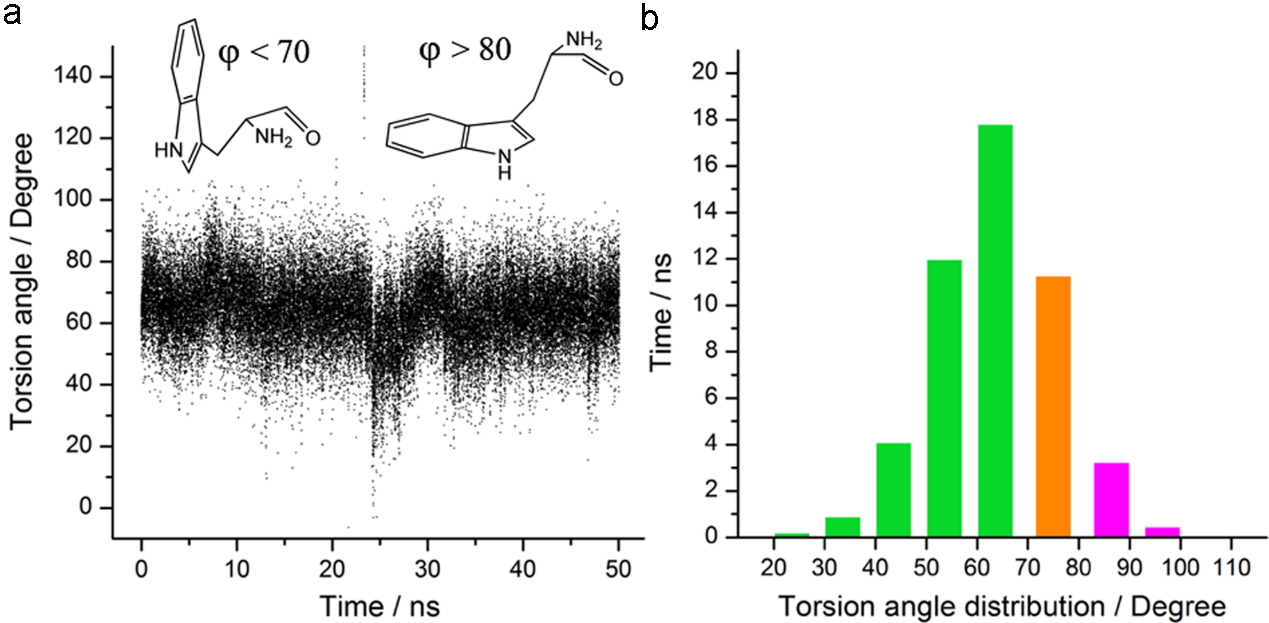
**

**Fig. S1. The dynamical change (A) and distribution (B) of torsion CA-CB-CG-CD2 in Trp21 during MD simulation.** The green columns were for the torsions ϕ<70, orange column was for the torsions 70<ϕ<80, and pink columns were for the torsions ϕ>80.

**
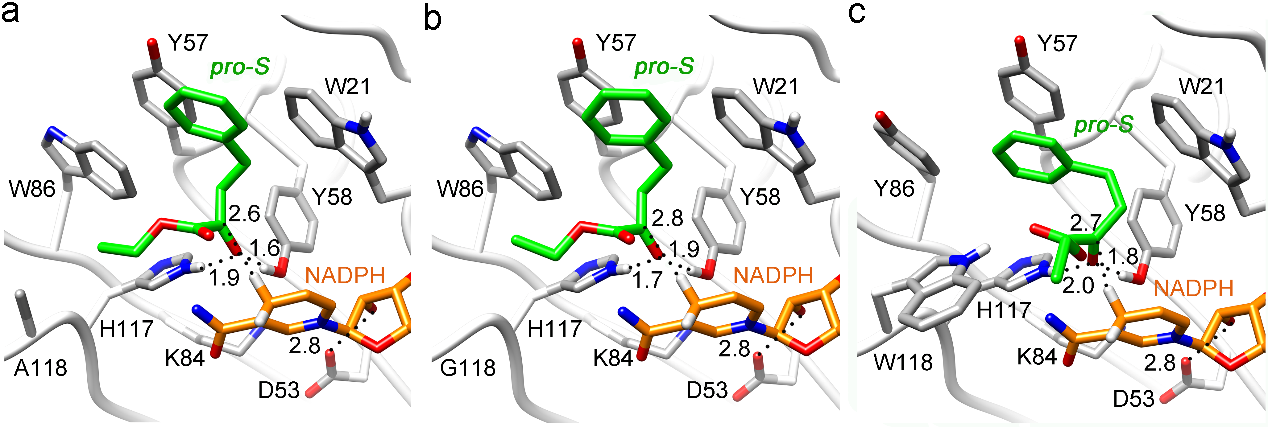
**

**Fig. S2. Binding conformations of EOPB with Tm1743 mutants W118A, W118G and W86Y.** Amino acids involved in EOPB coordination are shown as gray sticks. EOPB molecules with *pro-S* conformations are colored in green, and NADPH are colored in orange.

**
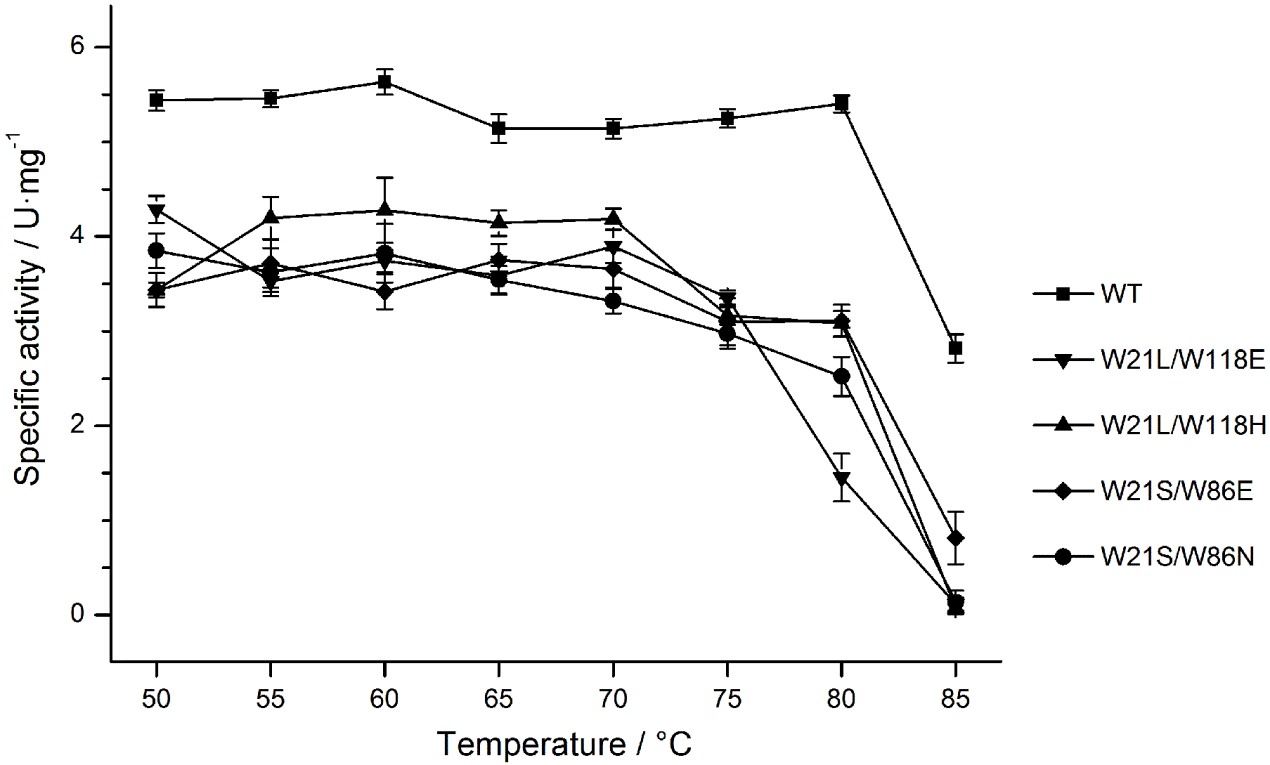
**

**Fig. S3.** **The thermal stability of the wild type and four Tm1743 double mutants.** Specific activity of the enzyme (U·mg^-1^) was plotted against temperatures. The standard errors were calculated from triplicate experiments.

**
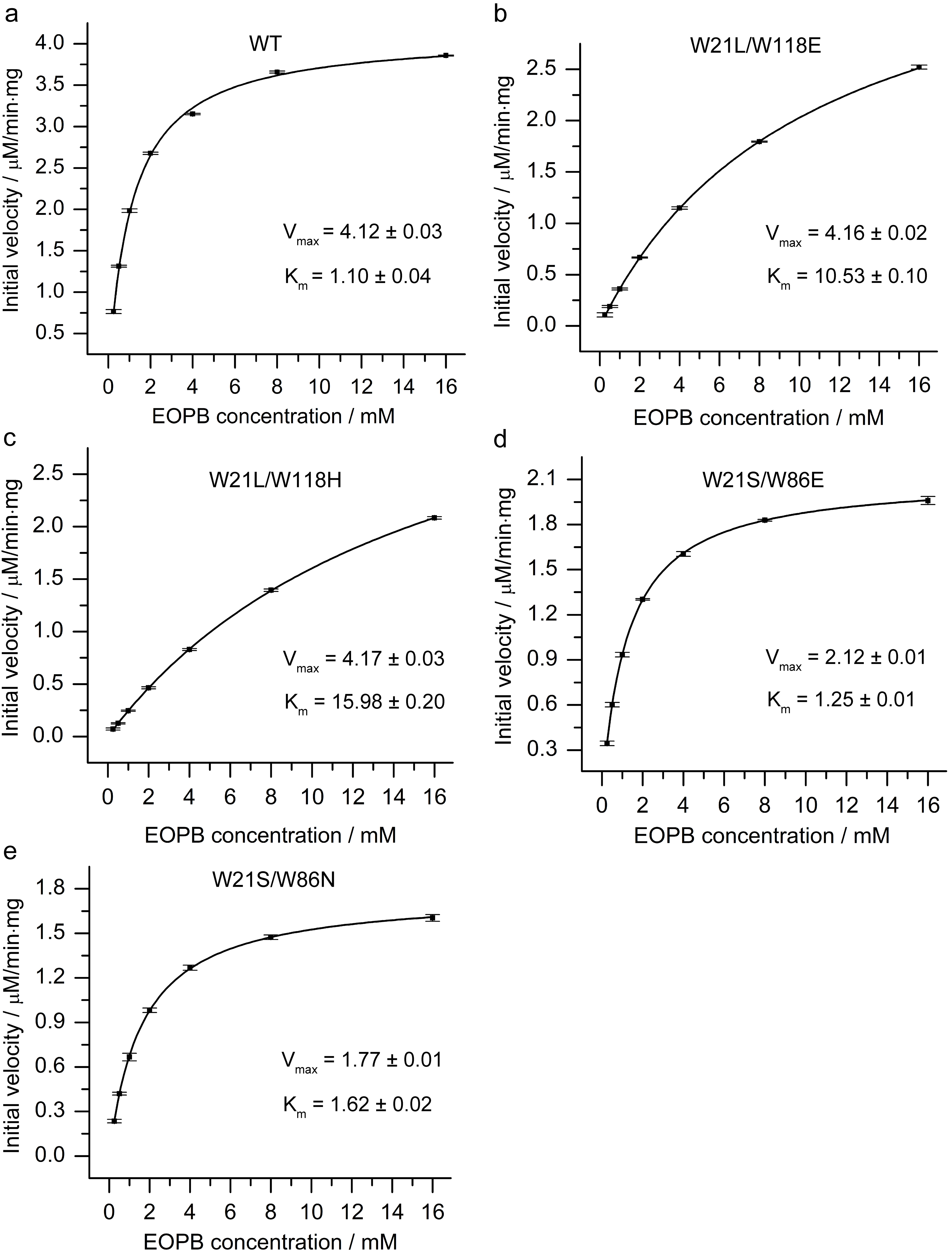
**

**Fig. S4. Kinetic parameters calculated using non-linear least squares fitting method based on the Michaelis-Menten equation V = V_max_·[S]/(K_m_+[S])**. *k_cat_* can be obtained based on the equation *k_cat_* = V_max_/[E]. [S] and [E] stand for the concentrations of the substrate and enzyme respectively. It is noted that the EOPB concentrations are not adequate to determine the kinetic parameters of mutants W21L/W118E and W21L/W118H, it is because further increase of EOPB concentration leads to aggregation of the reaction mixture. The plot was drawn with software Origin 8.0.
